# Study on the Multitarget Mechanism and Key Active Ingredients of Herba Siegesbeckiae and Volatile Oil against Rheumatoid Arthritis Based on Network Pharmacology

**DOI:** 10.1155/2019/8957245

**Published:** 2019-11-26

**Authors:** Xin Yang, Yahui Li, Runlin Lv, Haibing Qian, Xiangyun Chen, Chang Fu Yang

**Affiliations:** Guizhou University of Traditional Chinese Medicine, Guiyang 550025, China

## Abstract

**Background:**

Herba Siegesbeckiae (HS, Xixiancao in Chinese) is widely used to treat inflammatory joint diseases such as rheumatoid arthritis (RA) and arthritis, and its molecular mechanisms and active ingredients have not been completely elucidated.

**Methods:**

In this study, the small molecule ligand library of HS was built based on Traditional Chinese Medicine Systems Pharmacology (TCMSP). The essential oil from HS was extracted through hydrodistillation and analyzed by Gas Chromatography-Mass Spectrometer (GC-MS). The target of RA was screened based on Comparative Toxicogenomics Database (CTD). The key genes were output by the four algorithms' maximum neighborhood component (MNC), degree, maximal clique centrality (MCC), and stress in cytoHubba in Cytoscape, while biological functions and pathways were also analyzed. The key active ingredients and mechanism of HS and essential oil against RA were verified by molecular docking technology (Sybyl 2.1.1) in treating RA. The interaction between 6 active ingredients (degree ≥ 5) and CSF2, IL1*β*, TNF, and IL6 was researched based on the software Ligplot.

**Results:**

There were 31 small molecule constituents of HS and 16 main chemical components of essential oil (relative content >1%) of HS. There were 47 chemical components in HS. Networks showed that 9 core targets (TNF, IL1*β*, CSF2, IFNG, CTLA4, IL18, CD26, CXCL8, and IL6) of RA were based on Venn diagrams. In addition, molecular docking simulation indicated that CSF2, IL1*β*, TNF, and IL6 had good binding activity with the corresponding compounds (degree > 10).The 6 compounds (degree ≥ 5) of HS and essential oil had good interaction with 5 or more targets.

**Conclusion:**

This study validated and predicted the mechanism and key active ingredients of HS and volatile oil in treating RA. Additionally, this study provided a good foundation for further experimental studies.

## 1. Introduction

Rheumatoid arthritis (RA) is a chronic autoimmune disease that affects almost any joint of the human body [1]. IL-1*β* and IL-6 are known to be associated with the pathogenesis of RA [2]. IL-1*β* plays an exceedingly crucial role in the destruction of articular cartilage [3]. For increasing the release of collagenase and other proteolytic enzymes and inhibiting the cartilage cells for the synthesis of proteoglycans, IL-1β is able to stimulate the proliferation of the synovial and cartilage cells [4]. The primary functions of IL-6 are to stimulate the proliferation of B cells for the production of immunoglobulin and to stimulate the synovium for the production of the rheumatoid factor [5]. In addition, with the combination of soluble and membrane-bound tumour necrosis factor (TNF)-*α* [6], a firm immune complex is formed that inhibits the binding of TNF and its receptors [7], thereby further blocking the signal transduction of TNF and reducing the release of IL-1*β* and IL-6 for the control of inflammatory response [8]. In conclusion, the activities of these cytokines or receptors are closely related to the occurrence and development of RA [9].

Herba Siegesbeckiae (HS, Xixiancao in China) consists of the dry aerial parts of compositae plants including *Siegesbeckia orientalis* L., *S. pubescens* Makino, and *S. glabrescens* Makino. HS is one of the widely used traditional Chinese medicines that are prescribed by Chinese doctors for the treatment of inflammatory joint diseases such as RA and arthritis [10]. The main chemical constituents of HS consist of diterpenes, sesquiterpenes, and flavonoids, and pharmacological studies suggested that diterpenoids are the main antirheumatic constituents of HS [11]. Clinically, HS is mainly used in the treatment of RA, limb paralysis, muscle weakness, etc. [12]. Modern Clinical Pharmacological Research confirmed that HS probably reduces the levels of immunoglobulin G and circulating immune complexes [13]. This action of HS leads to a restricting effect on the cellular immunity and humoral immunity, stimulation of proliferation of the T cells, improvement of IL-2 activity, restriction in the activities of IL-1*β* and IL-6, and obstruction in the release of nitric oxide and TNF-*α* [14]. In short, it effectively adjusts the immune function and restricts the inflammatory mediators of the local tissues for diminishing the local inflammatory response, thereby achieving an excellent therapeutic purpose for RA [15].

**Figure 1:**
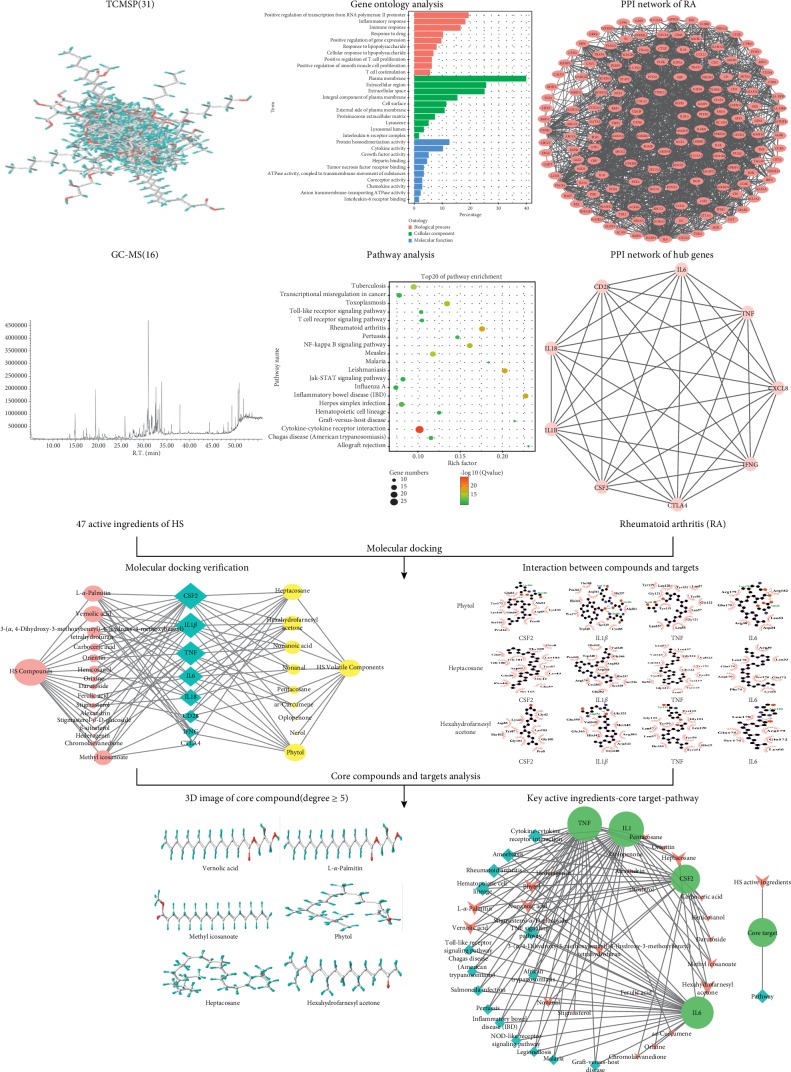
The framework of systematic analysis approach.

At present, the multidisciplinary crossover and the development of science and technology provide a more comprehensive platform and more powerful vehicle for evaluating the efficacy of traditional Chinese medicines (TCMs) [16]. In a sense, Computer-Aided Drug Design (CADD) is the important link between the TCMs and modern technology [17]. As a new field located on the general ideas of systems biology, it systematically and totally evaluates the pharmacological effects of multicomponent-target medicine [18, 19]. Due to the popularity of network pharmacology and molecular docking, several studies have used them to elucidate molecular mechanisms [20, 21]. Although several studies have indicated that HS can be effectively used for the treatment of inflammatory joint diseases such as RA and arthritis [11–13], there is still an urgent need for further clarification and description of its underlying mechanisms. Therefore, in this study, RA target network with hub genes was constructed by performing gene ontology enrichment analysis, pathway analysis, interaction analysis, and hub gene analysis of the RA targets. The key active ingredients and mechanism of HS and volatile oil are verified by molecular docking technology (Sybyl 2.1.1) in the treatment of RA. This study further validated and predicted the mechanism and key active ingredients of HS and volatile oil in the treatment of RA. A detailed fowchart of the network pharmacology-based study is shown in Figure 1.

## 2. Materials and Methods

### 2.1. Chemical Component Database Collection of Herba Siegesbeckiae

The information regarding the chemical candidates related to the HS was collected from the phytochemical database of Traditional Chinese Medicine Systems Pharmacology Database (TCMSP, http://ibts.hkbu.edu.hk/LSP/tcmsp.php) [22]. The TCMSP database provides information on ADME (absorption, distribution, metabolism, and excretion) properties such as drug-likeness, oral bioavailability (OB), blood-brain barrier (BBB), molecular weight (MW), Caco-2 permeability (Caco-2), H-bond donors (HBD), and H-bond acceptors (HBA) [23].

The chemical composition of HS essential oil was determined by employing the method of gas chromatography-mass spectrometry (GC-MS) [24]. The method of gas chromatography essential oil flame ionization detector (GC-FID) was performed on the HS essential oil using the Agilent GC (Model 6890/5975C) instrument equipped with the Agilent FB-5MSI capillary column (5% phenyl-methylpolysiloxane, 30 m × 0.25 mm × 0.25 *μ*m). Its initial temperature was 48°C (retained for 2 min), and then we heated it up to 220°C for 4 min. Thereafter, it was heated up to 310°C for 15 min and the heated sample was retained for 5 min. The total running time of the process was 56 min, whereas the temperature of the vaporization chamber was 250°C, the precolumn pressure was 7.65 psi, the carrier gas flew rate was 1.0 mL/min, the split ratio was 20 : 1, and the solvent delayed time was 5.0 min. The temperature of the ion source was 230°C, whereas the temperature of the quadrupole was 150°C. The EI source was the ion source, and the firing current had the value of 34.6 A. The values of electron energy and the multiplier voltage were 70 eV and 1659 V, respectively, with the interface temperature at 280°C, and the quality range was at 29–450 amu [25].

### 2.2. Rheumatoid Arthritis Target Identification by the Comparative Toxicogenomics Database

The RA targets were identified by the Comparative Toxicogenomics Database (CTD, http://ctdbase.org/). The CTD database is a robust, publicly available database for acquiring toxicogenomic information [26]. It provides manually curated core information about chemical diseases, chemical genes/protein interactions, and gene-disease relationships from peer-reviewed scientific literature. The candidate targets of RA were predicted using CTD with default parameters. The screening for potential target genes by the disease name “rheumatoid arthritis” or disease id “D015179” was done by selecting the target of “marker/memhanism (M)” or “thematic(T)” as the research object [27].

### 2.3. Functional and Pathway Enrichment Analysis

Gene ontology (GO) defines the concepts or classes that were used to describe the gene function and relationships [28]. GO_ MF (GO_molecular function), GO_BP (GO_biological progress) and GO_CC (GO_cell component) analyses were conducted for the RA target genes. The David database was also used to perform the pathway enrichment analysis with reference from the Kyoto Encyclopaedia of Genes and Genomes (KEGG) database website [29, 30]. GO and KEGG analyses were applied using the David database for identification of the targets. The cut-off criterion of *P* value was set as <0.05.

### 2.4. Protein-Protein Interaction (PPI) Network and Module Analysis

175 RA gene interactions based on STRING database (https://string-db.org/). The version 11.0 of STRING was employed to seek for the PPI data [31], with the species which were limited to “Homo sapiens,” and a confidence score of ≥0.4 was set as the threshold. Cytoscape (version 3.5.1) was used to visualize the PPI network of RA targets [32]. The Cytohubba plug-in was used to explore hub genes, and the top ten were generated using stress, degree, MNC, and MCC methods.

### 2.5. Molecular Docking

Molecular docking is a powerful computational tool which can predict the interaction energy between receptor and ligand. It determined the orientation of a ligand which would form the lowest energy complex within the receptor's binding pocket [33]. In this docking assay, eight human receptors were retrieved from Protein Data Bank (PDB): human TNF (hTNF, PDB ID: 2AZ5:2.1 Å), human IL6 (hIL6, PDB ID: 1ALU:1.9 Å), human IFNG (hIFNG, PDB ID: 1EKU:2.9 Å), human CTLA4 (hCTLA4, PDB ID: 3OSK:1.8 Å), human IL18 (hIL18, PDB ID: 3WO2:2.33 Å), human CD28 (hCD28, PDB ID: 1YJD:2.7 Å), human CSF2 (hCSF2, PDB ID: 5C7X:2.95:Å), and human IL1*β* (hIL1*β*, PDB ID: 1RWN:2.0 Å) [34]. In addition, the 3D structure of CXCL8 was not available. During this docking process, the threshold parameter was set at 0.5, and other parameters are of default value. The AMBER7 FF99 field was adopted to optimize energy and produce the active pocket by the Ligand model. Employ Sybyl 2.1.1 was used to evaluate the binding potential targets between RA targets and HS compounds. The Surflex–Dock score (total score) was expressed in −log10 (Kd) units [35].

### 2.6. Interaction between Compounds and Targets

This research was based on LigPlot + v.1.4.5. version [36], and the main ingredients of HS and CSF2, IL1*β*, TNF, and IL6 merg were preserved in PDB format, and at the same time, they were transferred to Ligplot software. The system is able to plot, in the same orientation, related sets of ligand-protein interactions. hydrogen bond and hydrophobic interaction are based on the HBPLUS program, and the structure is shown in the form of 2D, which is convenient for observation.

### 2.7. Target Identification and Network Construction

In order to interpret the pharmacological effect of active anti-RA compounds in HS, the C-T-P was constructed by connecting the active compounds, core targets, and their related pathway. The C-T-P network was visualized and analyzed by Cytoscape 3.5.1. In the network, the targets, compounds, and pathway were indicated by nodes, while edges represent the compound-target or target-pathway interactions.

## 3. Results

### 3.1. Chemical Composition Collection of HS and Volatile Oil

**Figure 2:**
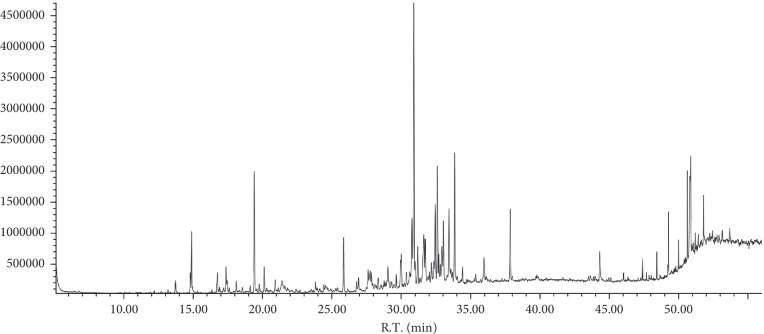
GC-MS chromatogram of compound HS essential oil.

The chemical constituents of HS were searched by TCMSP database, and there were 31 small molecule constituents of HS (Supplementary [Supplementary-material supplementary-material-1].). In this paper, the chemical components of compound HS essential oil were determined by GC-MS (Figure 2). The essential oil of HS was analyzed, and the composition is shown in supplementary [Supplementary-material supplementary-material-1]; 53 constituents were found which represent 63.52% of the total volatile oil HS. 16 major components (relative content >1%) for 49.728% of the total volatile oil HS are shown in Supplementary [Supplementary-material supplementary-material-1]. 31 chemical components screened from TCMSP data were excluded from the 16 volatile components of HS. There were 47 chemical components in HS.

### 3.2. RA Target Genes GO and Pathway Enrichment Analysis

**Figure 3:**
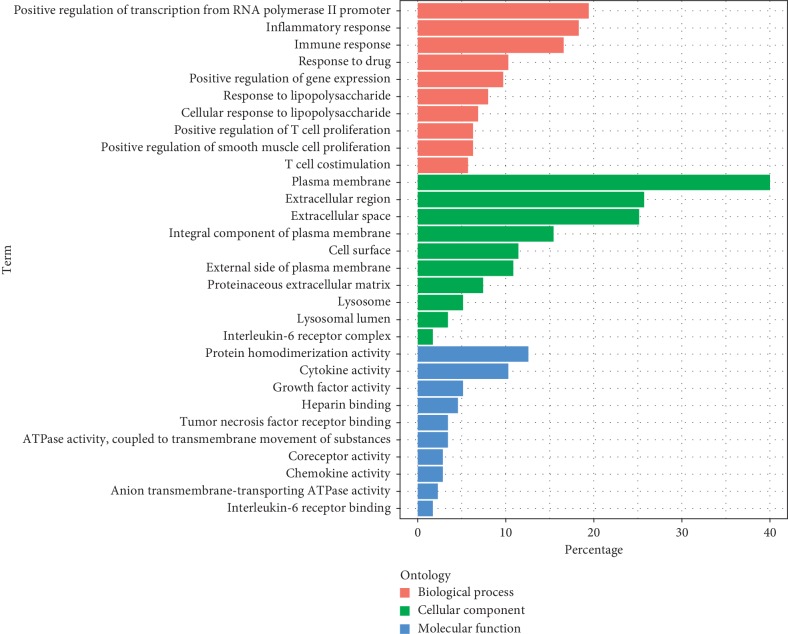
GO analysis of potential targets. The *Y*-axis shows the enrichment scores of these terms or the counts of targets, and the *X*-axis shows percentage-enriched GO categories of the target genes (*P* value < 0.01).

**Figure 4:**
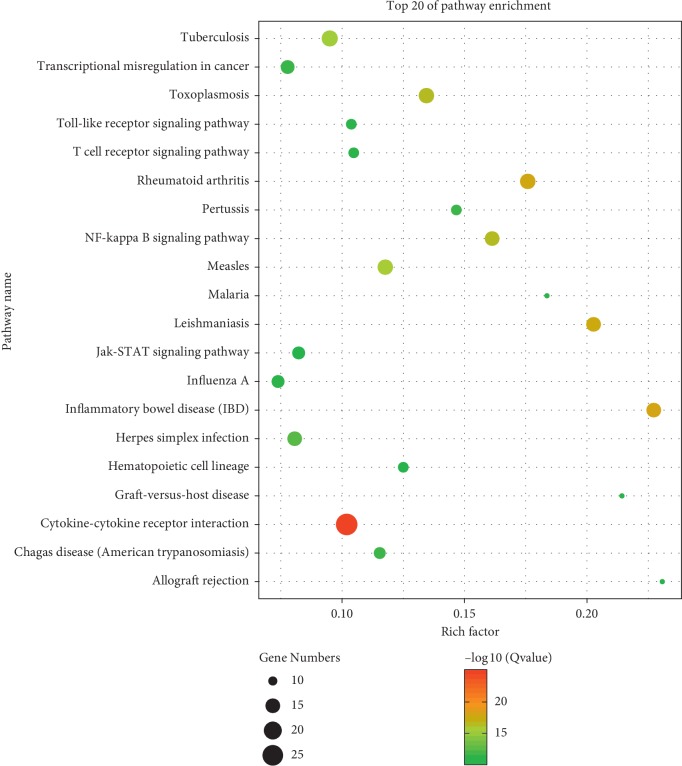
KEGG analysis of potential targets. In the picture, the *X*-axis represents the rich factor (*P* value < 0.01) and the *Y*-axis shows significantly enriched KEGG pathways of the target genes. The rich factor represents the ratio of the number of target genes belonging to a pathway to the number of all the annotated genes located in the pathway. The higher rich factor represents the higher level of enrichment. The size of the dot indicates the number of target genes in the pathway, and the color of the dot reflects the different *P* value ranges.

RA-related genes were selected from the CTD database, selecting the target of “marker/memhanism (M)” or “thematic (T)” as the research object, where the label “marker/memhanism (M)” indicates an experimentally validated gene, and the gene labeled “thematic (T)” indicates a therapeutic effect, for a total of 175 RA genes. In this study, GO Ontology and pathways of target protein participation were mapped from GO enrichment analysis and KEGG enrichment analysis. The targets of molecular function (Figure 3) were most related to cytokine activity. The cellular component targets are related to the extracellular space. The biological process was most related to the inflammatory response. The KEGG analysis involved a total of 23 pathways, most related to the cytokine-cytokine receptor action signaling pathway, toll-like receptor signaling pathway, and rheumatoid arthritis (Figure 4).

### 3.3. Protein-Protein Interaction (PPI) Network and Hub Gene of RA

**Figure 5:**
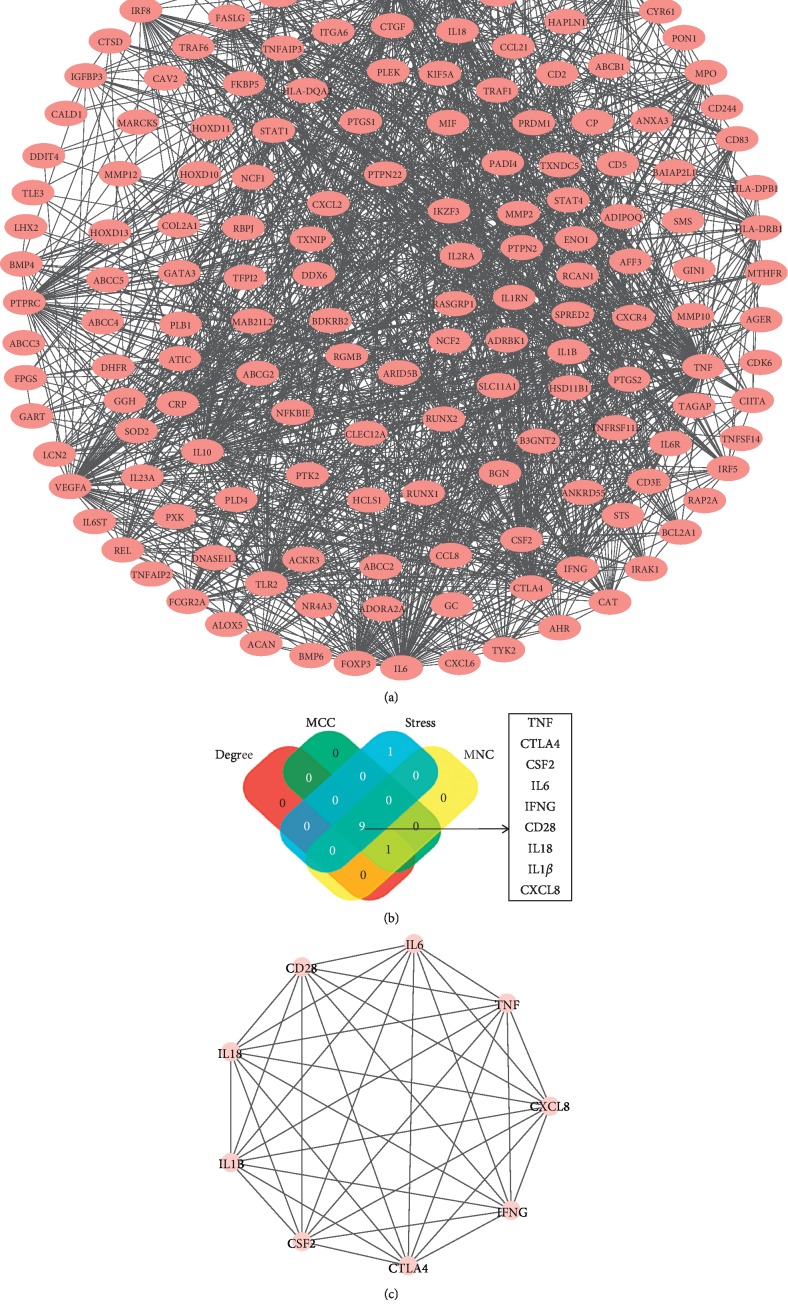
PPI network and hub clustering modules. (a) PPI networks of the RA targets. (b) Hub genes were screened from the PPI network using the MNC, MCC, degree, and stress methods. Overlapping datasets were visualized using Venn diagrams. (c) PPI network of hub genes.

175 RA genes entered into String database: a total of 174 nodes, number of edges of 1057, average node degree of 17.3, with PPI enrichment *P* value < 1.0*E* − 16. Many of these genes are operating together with others (Figure 5(a)). The RA target network was constructed based on coexpressed MNC, degree, MCC, and stress. Overlapping datasets were visualized using Venn diagrams (Figure 5(b)). 9 core targets (TNF, IL1*β*, CSF2, IFNG, CTLA4, IL18, CD28, CXCL8, and IL6) are shown in Figure 5(b). 9 core targets entered into String database: a total of 9 nodes, number of edges of 34, average node degree of 7.56, with PPI enrichment *P* value < 6.2*E* − 13 (Figure 5(c)).

### 3.4. Molecular Docking Verification

**Figure 6:**
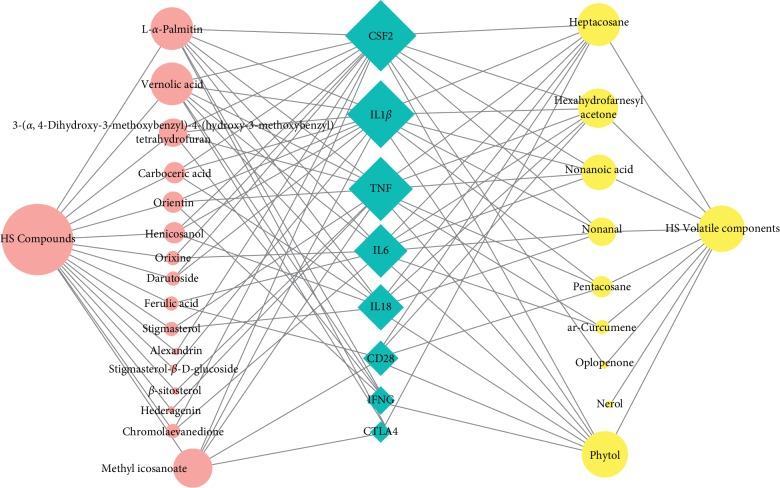
Yellow circles represent compounds in the volatile oil composition of HS. Red circles represent compounds in HS. Blue diamonds represent potential targets for RA. There is a positive proportional relationship between the node size and the degree.

These 8 core targets (TNF, IL1*β*, CSF2, IFNG, CTLA4, IL18, CD26, CXCL8, and IL6) were inputted into sybyl 2.1.1 for molecular docking verification (Figure 6). A total of 47 compound combinations were delivered into docking. The docking scores of most of them were larger than 5, which showed that they possessed good binding activity (Table 1). HS exerted treatment effects on RA by regulating 4 core targets (CSF2, IL1*β*, TNF, and IL-6), the 6 compounds (phytol, heptacosane, hexahydrofarnesyl acetone, vernolic acid, L-*α*-palmitin, and methyl icosanoate) of HS have good interaction with 5 or more targets (Table 2).

### 3.5. Analysis of Interaction between Core Compounds and Targets

The analysis of interaction between 6 active ingredients (degree ≧ 5) and CSF2, IL1*β*, TNF, and IL6 (degree > 10) was based on Ligplot1.4.5 software, and hydrogen bonding and hydrophobic interactions were outputted. Through the interaction verification analysis, it was found that the amino acid sites of Overnice acid, L-*α*-palmitin, methyl icosanoate, phytol, heptacosane, and hexahydrofarnesyl acetone interacted with CSF2, IL1*β*, TNF, and IL6 have great similarities, and these key amino acid sites may be closely related to the efficacy of HS (Tables 3 and 4).

### 3.6. Core Compounds and Target Analysis

**Figure 7:**
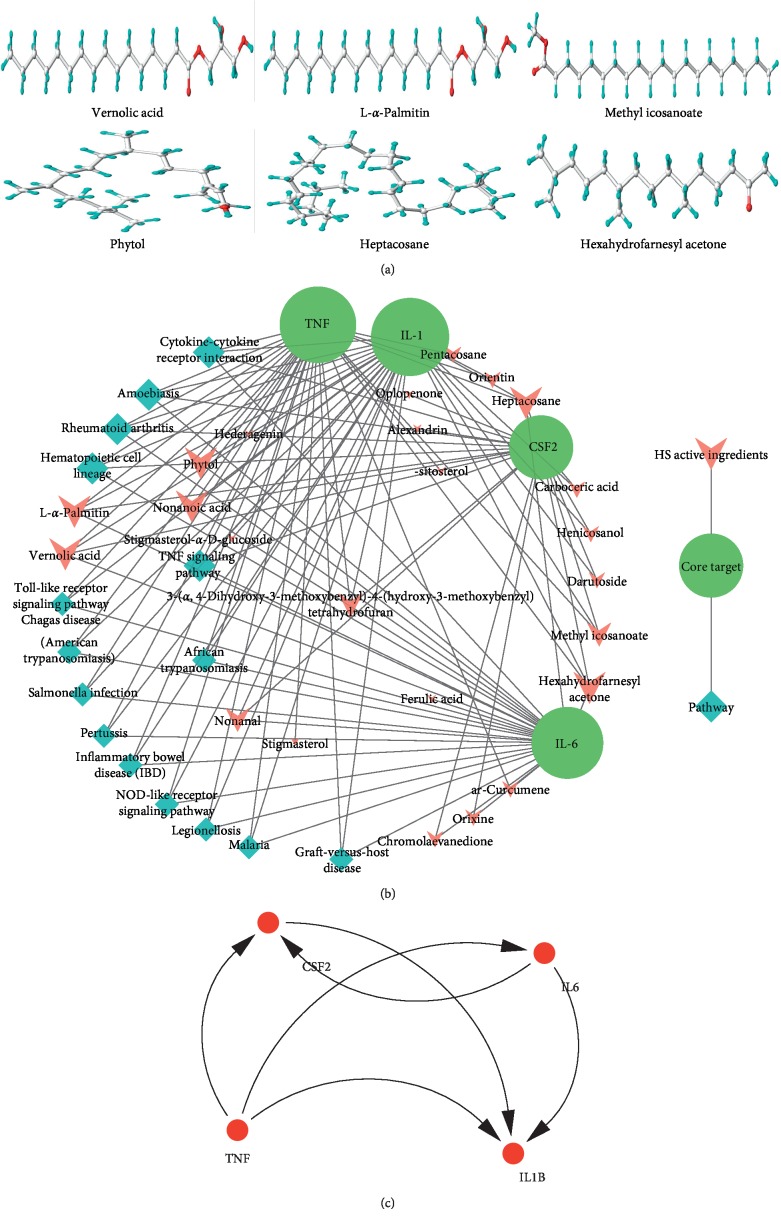
Linkage of target compounds and target genes. (a) 3D image of core compound (degree ≧ 5). (b) Compound-target-pathway network. (c) Protein-protein interaction network; the size of the nodes represents the value of the degree.

In the present work, for screening the potential active compounds from HS compounds with good activity, it requires OB ≧ 30%, DL ≧ 0.18, BBB ≧ -0.3, HBD < 10, HBA < 10, MW < 500 Da, Caco-2 ≧ -0.4, and AlogP > 5. Core compounds basically conform to Lipinski's rule of five (Table 5). The 3D image of core compound was shown in Figure 7(a).

Target fishing and C-T-P network construction. The results displayed that the most active compounds are linked with CSF2, IL1*β*, TNF, and IL-6, exhibiting extensive pharmacological effects of the bioactive ingredients. For instance, 10 and more than 10 chemical components of HS can be combined with CSF2, IL1*β*, TNF, and IL6, which involved in 15 pathways such as rheumatoid arthritis, TNF signaling pathway, and cytokine-cytokine receptor interaction (Figure 7(b)).4 core genes entered into String database, a total of 4 nodes, number of edges of 6, and average node degree of 3, with PPI enrichment *P* value < 0.0225 (Figure 7(c)). The coefficient of these four genes plays an important role in regulating the invasion of RA.

## 4. Discussion

This study based on network pharmacology and molecular docking technology revealed the structure-activity relationship of HS compound, involving 47 active ingredients, including terpenoids, glycosides, and volatile components. Through molecular docking, it was found that the chemical constituents of HS mainly regulated four targets (CSF2, IL1*β*, TNF, and IL6) to exert anti-RA effects. Overnice acid, L-*α*-palmitin, methyl icosanoate, phytol, heptacosane, and hexahydrofarnesyl acetone components of HS were mainly active in anti-RA. Through the interaction verification analysis, it was found that the amino acid sites of overnice acid, L-*α*-palmitin, methyl icosanoate, phytol, heptacosane, and hexahydrofarnesyl acetone interacted with CSF2, IL1*β*, TNF, and IL6 have great similarities. These key amino acid sites may be closely related to the efficacy of HS. The active components, RA targets, pathways, and interactions of HS were discussed in this study.

In recent years, it has been found that the active components in HS have anti-inflammatory, analgesic, antiallergic, and antitumor effects [37–39]. HS mainly contains diterpenoids, sesquiterpenes, flavonoids, and other chemical components [37, 40, 41]. This paper takes the active components of HS as the breakthrough point for a study since no systematic structure-function relationship study has been carried out at present. Through molecular docking, it was found that 25 of 47 active components of HS had good binding with core targets, and 6 of them had strong binding, mainly including terpenes, glycosides, fatty acids, volatile oil, and coumarins. These active components have been extracted and separated from HS, and pharmacological experiments on gouty arthritis and foot swelling have proved that some active components have anti-inflammatory and wind-dampness expelling effects [42–44], among which terpenoids have better anti-inflammatory functions [45].

The analysis of protein interaction showed that there was a correlation between CSF2, IL1*β*, TNF, and IL6, and 10 and more than 10 chemical components of HS can be combined with CSF2, IL1*β*, TNF, and IL6 (degree > 10). In particular, the combination with CSF2 and IL1*β* (degree = 16), the results further indicate that the prevention and treatment of RA by HS may play a role through multicomponent-multitarget combination. There have been reports that HS is mainly used to treat RA, confirming Modern Clinical Pharmacological Research [10] and HS probably reduces the level of immunoglobulin G and circulating immune complex (CIC) [46], making inhibitory effect on cellular immunity and humoral immunity [10], stimulating the proliferating function of T cells, improving the activity of IL-2, and inhibiting the activity of IL-1*β* and IL-6, also inhibiting the release of nitric Oxide and TNF-*α* [47]. In short, it effectively adjusts the immune function and inhibits the inflammatory mediators of local tissue for retarding the local inflammatory response, which achieves the excellent therapeutic purpose on RA [48]. The results in this study were corresponded with the report of literatures completely. In conclusion, CSF2, IL1*β*, TNF, and IL6 may be a key target for HS in the treatment of RA.

More and more evidences have shown that the pathophysiological mechanism of RA is very complex, and there are various biological processes and signal pathways involved in the process of RA damage [49]. 175 RA targets screened in this study mainly take part in the release of inflammatory cytokines and proinflammatory factors by pathways such as TNF, NF-κB, and toll-like receptor signaling pathway [50, 51]. According to the constructed network model of “active component-core target-pathway,” HS may intervene the inflammation and immune pathways to reduce the release of inflammatory cytokines and proinflammatory factors through “multicomponent-multitarget” or “multicomponent-single-target.” Increasing pharmacological experiments have confirmed that the extract and monomer components of HS have an influence on the anti-inflammatory and analgesic effects of cytokines through TNF-*α*, Wnt/*β*-catenin, JNK, and other signaling pathways [5, 52]. To sum up, HS may have significant potential to treat RA by a combination of multicomponents, multitargets, and multipathways.

Through network pharmacology combined with molecular docking technology, the possible active components and molecular mechanisms of HS for treating RA were systematically screened in this study to provide a new breakthrough point for the treatment of RA. However, its rationality is only preliminarily explained in this study that still has some limitations.

## 5. Conclusions

In the present study, we found 31 small molecule constituents of HS and 16 main chemical components of essential oil (relative content >1%) of HS, involving 47 active ingredients, including terpenoids, glycosides, and volatile components. HS exerted treatment effects on RA by regulating 4 core targets (CSF2, IL1*β*, TNF, and IL6), which involved in 15 pathways such as rheumatoid arthritis, TNF signaling pathway, and cytokine-cytokine receptor interaction. The 6 compounds (phytol, heptacosane, hexahydrofarnesyl acetone, vernolic acid, L-*α*-palmitin, and methyl icosanoate) of HS have good interaction with 5 or more targets. The analysis of protein interaction showed that there was a correlation between CSF2, IL1*β*, TNF, and IL6. The coeffection of these four genes plays an important role in regulating the invasion of RA. 10 and more than 10 chemical components of HS can be combined with CSF2, IL1*β*, TNF, and IL6 (degree > 10). In particular, the combination with CSF2 and IL-1*β* (degree = 16), the results further indicate that the prevention and treatment of RA by HS may play a role through multicomponent-multitarget combination.

In this study, the chemical components screened in this study included the main chemical components of HS, and also added volatile components, network pharmacology, and molecular docking were used to screen out key targets and active ingredients. Confirmed by the results, the network pharmacology method wonderfully validated and forecasted the molecular mechanism of HS in RA at a system level, which not only might build the solid foundation to deepen understanding of the mechanisms of HS and other anti-inflammatory TCMs but also facilitated the widespread application of HS in treating RA. No matter how, the results from our research are based on computational analysis, and further experiments are needed to verify these hypotheses.

## Figures and Tables

**Table 1:** The docking information of 8 targets with the corresponding compounds.

Target	HS volatile components	Score	HS compound	Score
TNF	Phytol	7.5	Vernolic acid	7.6
	Hexahydrofarnesyl acetone	7.1	3-(*α*,4-Dihydroxy-3-methoxybenzyl)-4-(hydroxy-3-methoxybenzyl) tetrahydrofuran	5.9
	Heptacosane	6.6	L-*α*-Palmitin	5.8
	Pentacosane ar-curcumene	6.2	Methyl icosanoate	5.6
	Nonanoic acid	6.2	Orientin	5.2
		5.0	*β*-Sitosterol	5.2
			Hederagenin	5.0
			Stigmasterol	5.0

IL1*β*	Phytol	7.2	Carboceric acid	10.8
	Heptacosane	6.8	L-*α*-Palmitin	9.4
	Pentacosane	6.4	Vernolic acid	7.9
	Hexahydrofarnesyl acetone	5.8	Methyl icosanoate	7.4
	Nonanoic acid	5.2	Henicosanol	7.2
	Nonanal	5.1	3-(*α*,4-Dihydroxy-3-methoxybenzyl)-4-(hydroxy-3-methoxybenzyl) tetrahydrofuran	5.6
			Darutoside	5.6
			Orientin	5.3
			Stigmasterol-*β*-D-glucoside	5.0

CSF2	Heptacosane	6.8	Carboceric acid	7.8
	Phytol	5.9	Methyl icosanoate	7.3
	Nonanal	5.6	Darutoside	6.9
	Nonanoic acid	5.4	Henicosanol	6.7
	Oplopenone	5.3	L-*α*-Palmitin	6.4
	Hexahydrofarnesyl acetone	5.2	Vernolic acid	6.3
			3-(*α*,4-Dihydroxy-3-methoxybenzyl)-4-(hydroxy-3-methoxybenzyl) tetrahydrofuran	5.7
			Alexandrin	5.7
			Orixine	5.6
			Chromolaevanedione	5.4

IFNG	Phytol	5.4	Vernolic acid	6.7
			L-*α*-Palmitin	6.6
			Orientin	5.6
			3-(*α*,4-Dihydroxy-3-methoxybenzyl)-4-(hydroxy-3-methoxybenzyl) tetrahydrofuran	5.2

CTLA4	Heptacosane	5.2	L-*α*-Palmitin	5.1
			Methyl icosanoate	5.0
			Vernolic acid	5.0

IL18	Nonanal	6.4	L-*α*-Palmitin	5.5
	Hexahydrofarnesyl acetone	5.1	Stigmasterol	5.4
	Phytol	5.0	Vernolic acid	5.3
	Heptacosane	5.0	Carboceric acid	5.3
			Henicosanol	5.1

CD28	Heptacosane	6.4	Ferulic acid	5.2
	Hexahydrofarnesyl acetone	6.2	Methyl icosanoate	5.0
	Phytol	5.8		
	Pentacosane	5.6		

IL6	Hexahydrofarnesyl acetone	6.0	Vernolic acid	7.2
	ar-Curcumene	5.8	L-*α*-Palmitin	6.5
	Nonanoic acid	5.6	Chromolaevanedione	5.2
	Nonanal	5.6	Ferulic acid	5.2
	Heptacosane	5.5	Orixine	5.0
	Phytol	5.3		

**Table 2:** Chemical composition and target molecule docking.

Tagret	Degree	Volatile oil (GS-MS)	Degree	Compounds (TCMSP)	Degree
CSF2	16	Phytol	7	Vernolic acid	7
IL1*β*	16	Heptacosane	7	L-*α*-Palmitin	7
TNF	14	Hexahydrofarnesyl acetone	6	Methyl icosanoate	5
IL6	11	Nonanoic acid	4	3-(*α*,4-Dihydroxy-3-methoxybenzyl)-4-(hydroxy-3-methoxybenzyl) tetrahydrofuran	4
IL18	9	Nonanal	4	Orientin	3
CD28	6	Pentacosane	3	Henicosanol	3
IFNG	6	ar-curcumene	2	Orixine	3
CTLA4	4	Oplopenone	1	Carboceric acid	3
		Nerol	1	Chromolaevanedione	2
				Ferulic acid	2
				Stigmasterol	2
				Darutoside	2
				*β*-Sitosterol	1
				Hederagenin	1
				Stigmasterol-*β*-D-glucoside	1
				Alexandrin	1

**Table 3:** The 2D protein-ligand interaction (hydrogen bond).

Compounds	Hydrogen bond			
	CSF2	IL-1*β*	TNF	IL-6
Overnice acid	Gly100 Tyr87	Ser236 Arg341 Arg179	—	Arg182
L-*α*-Palmitin	Lys166	Val348 Arg352	Gly121 Tyr151	Arg30 Arg179 Arg182
Methyl icosanoate	Lys166	—	Tyr151	Arg182
Phytol	Leu104 Thr105	Arg179 Ser236 Gln283	Ser60	Leu178 Asp26
Heptacosane	—	—	—	—
Hexahydrofarnesyl acetone	—	Arg352	Tyr151	Arg182

**Table 4:** The 2D protein-ligand interaction (hydrophobic interactions).

Compounds	Hydrophobic interactions			
	CSF2	IL-1*β*	TNF	IL-6
Overnice acid	Lys166 Ser165 Tyr172 Glu83 Ala84 Pro40 Lys103 Asp85 Lys43 Gly42 Leu104 Gly44	Trp340 Arg383 Pro343 His342 Ser339 His237 Cys285	Leu57 Leu120 Gly121 Tyr119 Tyr151 Ser60 Tyr59 Ile155	Lys66 Glu172 Phe74 Ser176 Arg179 Gln175 Leu178

L-*α*-Palmitin	Pro41 Lys43 Val189 Gly42 ASP85 Ser165 Tyr172 Pro40 Lys103 Lys39 Gln38 Gln39 Ala40	Arg383 His342 Met345 Glu355 Gly351 Arg341 Trp340	Tyr151 Tyr59 Leu120 Tyr119 Ser60 Glu148 Ser60 His15 Val17	Ser176 Gln175 Asp26 Leu178 Phe74

Methyl icosanoate	Ala84 Ser165 Tyr172 Pro40 Gly42 Lys43 Leu108 Pro41 Val189 Gln38 Gln39 Lys103 Asp85 Glu83	Arg383 Trp340 Gly346 Ser347 Glu355 Gln358 Ile354 Gly351 Val348 His342 Pro343 Arg341	Leu57 Ser60 Tyr119 Ser95 Gln125 Leu55 Leu120 Tyr59 Gly121 Gly122 Gln61	Leu178 Arg179 Lys171 Gln175 Arg40

Phytol	Glu83 Tyr172 Lys166 Gln38 Ser165 Pro164 Pro40 Asp85 Lys103 Ala84	Thr180 Pro343 His342 Pro177 Trp340 Ser339 Cys285 Ala284 His237 Arg341	Tyr119 Leu120 Leu57 Tyr151 Gly121 Tyr59 Gly122 Leu55 Leu157	Arg179 Gln175 Arg30 Leu33 Asp34 Arg182

Heptacosane	Gln6 Gly100 Gly101 Asp85 Gln38 Pro40 Ser165 Gln39 Gly42 Lys43 Tyr87 Lys103 Thr102 Pro8	His342 Pro343 Trp340 Arg341 Arg179 Cys285 Gln283 Ser339 Ser236 His237 Arg383 Gly346 Val348	Leu57 Leu157 Val123 Gly121 Tyr59 Tyr119 Tyr151	Arg30 Leu178 Gln175 Arg179 Phe74 Lys66 Ser176 Glu172 Leu33

Hexahydrofarnesyl acetone	Lys43 Asp85 Tyr87 Thr102 Gly101 Pro8 Gl100 Lys103 GLY42	Glu355 Val348 Gly346 His342 Trp340 Arg341 Arg383 Met345 Gly351	Gly122 Tyr59 Leu57 Ile57 Ile155 Tyr151 His15 Leu120 Tyr119	Leu178 Gln175 Ser176 Lys66 Glu172 Arg179

**Table 5:** Physical and chemical properties of key chemical constituents.

Chemical compound	MW	AlogP	HBD	HBA	OB%	Caco-2	BBB	DL
Overnice acid	296.50	5.40	1	3	37.63	0.72	0.18	0.19
L-*α*-Palmitin	330.57	5.57	2	4	26.66	0.30	−0.42	0.22
Methyl icosanoate	326.63	8.44	0	2	15.79	1.43	1.12	0.22
Phytol	296.60	7.34	1	1	33.82	1.23	0.85	0.13
Heptacosane	380.83	12.69	0	0	8.18	1.88	1.80	0.36
Hexahydrofarnesyl acetone	268.54	6.20	0	1	6.67	1.50	1.44	0.10

## Data Availability

The data used to support the findings of this study are included within the supplementary information files.

## Supplementary Materials

Supplementary Materials Supplementary materials contain four tables. Supplementary Table S1: physical and chemical properties of HS chemical constituents. Supplementary Table S2: chemical composition in volatile oils of HS. Supplementary Table S3: major components (relative content >1%) in volatile oils of HS. Supplementary Table S4: RA target genes. Supplementary Figure S1: structure of interaction between main components and target point.
