# Supplementary material for: Study on the Multitarget Mechanism and Key Active Ingredients of Herba Siegesbeckiae and Volatile Oil against Rheumatoid Arthritis Based on Network Pharmacology
Source: Evid Based Complement Alternat Med. 2019 Nov 26;2019:8957245. doi: 10.1155/2019/8957245 (PMC6899322; doi:10.1155/2019/8957245)
Supplement: Supplementary Materials — contain four tables. Supplementary Table S1: physical and chemical properties of HS chemical constituents. Supplementary Table S2: chemical composition in volatile oils of HS. Supplementary Table S3: major components (relative content >1%) in volatile oils of HS. Supplementary Table S4: RA target genes. Supplementary Figure S1: structure of interaction between main components and target point. [file 8957245.f1.doc]

Table S1 Physical and chemical properties of SH chemical constituents

| NO. | Compounds | MW | AlogP | HBA | HBD | OB% | DL |
| --- | --- | --- | --- | --- | --- | --- | --- |
| 1  2  3  4  5  6  7  8  9  10  11  12  13  14  15  16  17  18  19  20  21  22  23  24  25  26  27  28  29  30  31 | L-*α*-Palmitin  Cumalic acid  Alexandrin  Hederagenin  Stigmasterol  Succinic acid  *β*-sitosterol  Ferulic acid  Methyl icosanoate  3-(α,4-Dihydroxy-3-methoxybenzyl)-4-(hydroxy-3-methoxybenzyl) tetrahydrofuran  (1R)-1-[(2S,4aR,4bS,7R,8aS)-7-hydroxy-2,4b,8,8-tetramethyl-4,4a,5,6,7,8a,9,10-octahydro-3H-phenanthren-2-yl]ethane-1,2-diol  Darutoside  Epsilon-cadinene  Euscaphic acid  Germacrane D  15α-Hydroxy-ent-kaur-16-en-19-oic acid  Orientin  Vernolic acid  Coronaridine  Henicosanol  (1R)-1-[(2S,4aR,4bS,6S,8R,8aS)-6-hydroxy-2,4b,8-trimethyl-8-methylol-4,4a,5,6,7,8a,9,10-octahydro-3H-phenanthren-2-yl]ethane-1,2-diol  Orixine  Siegesesteric acid II  Siegesmethyletheric acid  Soladulcidin  Stigmasterol-β-D-glucoside  Chromolaevanedione  Darutigenol B  δ-amorphene  Darutoside  Carboceric acid | 330.57  140.1  576.95  414.79  412.77  118.1  414.79  194.2  326.63  360.44  322.54  322.54  204.39  488.78  210.45  318.5  448.41  296.5  338.49  312.65  338.54  335.39  348.58  334.55  415.73  484.7  222.36  322.54  204.39  574.93  410.81 | 5.57  -0.11  6.34  8.08  7.64  -0.41  8.08  1.62  8.44  2.46  3.00  3.00  4.85  4.35  6.03  3.78  -0.32  5.4  4.06  8.73  1.77  2.08  4.55  4.20  4.62  1.25  1.87  2.86  4.94  5.89  11.39 | 2  1  4  1  1  2  1  2  0  3  3  3  0  4  0  2  8  1  1  1  4  2  1  1  2  6  0  2  0  4  1 | 4  4  6  1  1  4  1  4  2  6  3  3  0  5  0  3  11  3  3  1  4  7  3  3  3  8  2  3  0  6  2 | 26.66  43.10  20.63  36.91  43.83  29.62  36.91  39.56  15.79  5.53  46.7  29.93  16.41  17.32  15.46  58.73  1.79  37.63  34.97  11.86  28.38  18.71  51.98  60.72  17.08  4.08  21.99  16.74  17.95  21.32  13.96 | 0.22  0.03  0.63  0.75  0.76  0.01  0.75  0.06  0.22  0.40  0.31  0.31  0.08  0.71  0.06  0.38  0.75  0.19  0.68  0.19  0.35  0.35  0.48  0.43  0.8  0.85  0.09  0.38  0.08  0.63  0.44 |

Table S2 Chemical composition in volatile oils of SH

| Compound name | RI | Mole. Formula | MW Content (%) | |
| --- | --- | --- | --- | --- |
| 2-Methylbutanoic acid  1-Hexanol  1-Heptanol  1-Octen-3-ol  1,8-Cineole  Benzeneacetaldehyde  1-Octanol  Linalool  Nonanal  Camphor  Nerol oxide  (E)-2-Nonenal  (-)-Borneol  Nonanol  L-(-)-Menthol  4-Terpineol  α-Terpineol  Decanal  Nerol  Methyl thymyl ether  Pulegone  trans-Geraniol  1-Decanol  Nonanoic acid  Carvacrol  2,4-Decadienal  Eugenol  *α*-Copaene  Decanoic acid  trans-Caryophyllene  *β*-Cubebene  Geranyl acetone  *α*-Humulene  *α*-amorphene  ar-Curcumene  *β*-Ionone  *α*-Muurolene  *α*-Calacorene  Spathulenol  Caryophyllene oxide  Salvial-4(14)-en-1-one  Oplopenone  caryophylla-4(12),8(13)-dien-5β-ol  T-Muurolol  Hexahydrofarnesyl acetone  Phytol  Docosane  Tricosane  Tetracosane  Pentacosane  Hexacosane  Heptacosane  Nonacosane  Total | 868  869  970  979  1035  1043  1070  1103  1104  1143  1153  1162  1173  1171  1172  1182  1189  1203  1228  1236  1237  1255  1272  1293  1299  1309  1356  1377  1382  1414  1391  1455  1462  1485  1486  1496  1499  1523  1576  1582  1595  1606  1632  1634  1845  2116  2201  2304  2402  2503  2602  2705  2903 | C_5_H_10_O_2_  C_6_H_14_O  C_7_H_16_O  C_8_H_16_O  C_10_H_18_O  C_8_H_8_O  C_8_H_18_O  C_10_H_18_O  C_9_H_18_O  C_10_H_16_O  C_10_H_16_O  C_9_H_16_O  C_10_H_18_O  C_9_H_20_O  C_10_H_20_O  C_10_H_18_O  C_10_H_18_O  C_10_H_20_O  C_10_H_18_O  C_11_H_16_O  C_10_H_16_O  C_10_H_18_O  C_10_H_22_O  C_9_H_18_O_2_  C_10_H_14_O  C_10_H_16_O  C_10_H_12_O_2_  C_15_H_24_  C_10_H_20_O_2_  C_15_H_24_  C_15_H_24_  C_13_H_22_O  C_15_H_24_  C_15_H_24_  C_15_H_22_  C_13_H_20_O  C_15_H_24_  C_15_H_20_  C_15_H_24_O  C_15_H_24_O  C_15_H_24_O  C_15_H_24_O  C_15_H_24_O  C_15_H_26_O  C_18_H_36_O  C_20_H_40_O  C_22_H_46_  C_23_H_48_  C_24_H_50_  C_25_H_52_  C_26_H_54_  C_27_H_56_  C_29_H_60_ | 102  102  116  128  154  120  130  154  142  152  152  140  154  144  156  154  154  156  154  164  152  154  158  158  150  152  164  204  172  204  204  194  204  204  202  192  204  200  220  220  220  220  220  222  268  296  310  324  338  352  366  380  408 | 0.018  0.043  0.037  0.043  0.106  0.095  0.648  0.799  2.419  0.105  1.063  0.236  0.217  0.927  0.488  0.160  0.466  0.214  5.294  0.141  0.405  0.211  0.540  1.493  0.136  0.163  0.436  0.231  0.274  2.638  0.179  0.386  0.474  0.994  1.381  0.545  0.515  0.645  2.684  13.079  1.602  1.945  3.470  4.996  3.052  1.626  0.202  0.508  0.684  1.344  0.544  1.642  0.977  63.52 |

Table S3 Major components (Relative Content＞1%) in volatile oils of SH

| NO. | Compound name Mole. Formula MW Relative Content (%) | | | |
| --- | --- | --- | --- | --- |
| 1  2  3  4  5  6  7  8  9  10  11  12  13  14  15  16 | Nonanal  Nerol oxide  Nerol  Nonanoic acid  trans-Caryophyllene  ar-Curcumene  Spathulenol  Caryophyllene oxide  Salvial-4(14)-en-1-one  Oplopenone  caryophylla-4(12),8(13)-dien-5β-ol  T-Muurolol  Hexahydrofarnesyl acetone  Phytol  Pentacosane  Heptacosane | C_9_H_18_O  C_10_H_16_O  C_10_H_18_O  C_9_H_18_O_2_  C_15_H_24_  C_15_H_22_  C_15_H_24_O  C_15_H_24_O  C_15_H_24_O  C_15_H_24_O  C_15_H_24_O  C_15_H_26_O  C_18_H_36_O  C_20_H_40_O  C_25_H_52_  C_27_H_56_ | 142  152  154  158  204  202  220  220  220  220  220  222  268  296  352  380 | 2.419  1.063  5.294  1.493  2.638  1.381  2.684  13.079  1.602  1.945  3.470  4.996  3.052  1.626  1.344  1.642 |

Table S4 RA Target genes

| NO. | Gene | Gene id | Disease Name | Direct Evidence |
| --- | --- | --- | --- | --- |
| 1  2  3  4  5  6  7  8  9  10  11  12  13  14  15  16  17  18  19  20  21  22  23  24  25  26  27  28  29  30  31  32  33  34  35  36  37  38  39  40  41  42  43  44  45  46  47  48  49  50  51  52  53  54  55  56  57  58  59  60  61  62  63  64  65  66  67  68  69  70  71  72  73  74  75  76  77  78  79  80  81  82  83  84  85  86  87  88  89  90  91  92  93  94  95  96  97  98  99  100  101  102  103  104  105  106  107  108  109  110  111  112  113  114  115  116  117  118  119  120  121  122  123  124  125  126  127  128  129  130  131  132  133  134  135  136  137  138  139  140  141  142  143  144  145  146  147  148  149  150  151  152  153  154  155  156  157  158  159  160  161  162  163  164  165  166  167  168  169  170  171  172  173  174  175 | IFNG  IL1*β*  IL6  PTGS2  IL10  TLR2  CXCL8  CXCR4  SOD2  VEGFA  MPO  PTGS1  IL23A  CAT  STAT1  ACKR3  STAT4  ABCB1  BCL2A1  IGFBP3  TXNIP  FASLG  MARCKS  MMP2  CXCL2  IL18  CSF2  CTGF  ABCC4  ABCG2  DDIT4  FKBP5  TNFAIP2  CTSD  TNFSF14  IRAK1  TFPI2  COL2A1  ENO1  CYR61  MMP10  LCN2  NDUFA4L2  IL6ST  CP  ADORA2A  TMPO  SMS  HSD11B1  TNFAIP3  ANXA3  GSDME  PON1  NR4A3  CDK6  REL  CD40  BMP4  ABCC2  LY96  NCF1  CRP  CD83  TRAF6  TLE3  FOXP3  GATA3  GART  GPRC5A  ALOX5  STS  DHFR  NCF2  CAV2  IL2RA  ARID5B  F2  PTK2  RCAN1  CD2  MMP12  RUNX2  MIF  ABCC3  HLA-DRB1  PTPRC  RAB8A  B3GNT9  ADIPOQ  NFKBIE  RUNX1  RASGRP1  TNFRSF14  ACAN  CD28  AHR  CCR6  HCLS1  GGH  RAP2A  CCL8  BDKRB2  PLEK  MGARP  CXCL6  TRAF1  ABCC5  IRF5  SLC11A1  LHX2  DDX6  DNASE1L3  PRKCQ  CD5  MTHFR  AGER  ITGA6  BMP6  SLC25A12  TYK2  B3GNT2  SLC22A4  GC  PSG5  ATIC  TXNDC5  PTPN2  IL2RB  RGMB  IRF8  PXK  CD3E  FPGS  POU3F1  CIITA  CALD1  BAIAP2L1  GRK6  HOXD11  BGN  ST6GALNAC5  HLA-DPB1  PTPN22  SPRED2  TAGAP  GIN1  GDF5  CCL21  AFF3  FCGR2A  HAPLN1  CLEC12A  PRDM1  NFKBIL1  HOXD13  NOX2  MAB21L2  RBPJ  GRK2  MMEL1  CTLA4  ANKRD55  PLB1  IKZF3  CD244  PADI4  HOXD10  BLK  PLD4  HLA-DQA2  KIF5A  TNF  IL1RN  IL6R  IFNG | 3458  3553  3569  5743  3586  7097  3576  7852  6648  7422  4353  5742  51561  847  6772  57007  6775  5243  597  3486  10628  356  4082  4313  2920  3606  1437  1490  10257  9429  54541  2289  7127  1509  8740  3654  7980  1280  2023  3491  4319  3934  56901  3572  1356  135  7112  6611  3290  7128  306  1687  5444  8013  1021  5966  958  652  1244  23643  653361  1401  9308  7189  7090  50943  2625  2618  9052  240  412  1719  4688  858  3559  84159  2147  5747  1827  914  4321  860  4282  8714  3123  5788  4218  84752  9370  4794  861  10125  8764  176  940  196  1235  3059  8836  5911  6355  624  5341  84709  6372  7185  10057  3663  6556  9355  1656  1776  5588  921  4524  177  3655  654  8604  7297  10678  6583  2638  5673  471  81567  5771  3560  285704  3394  54899  916  2356  5453  4261  800  55971  2870  3237  633  81849  3115  26191  200734  117289  54826  8200  6366  3899  2212  1404  160364  639  4795  3239  100169685  10586  3516  156  79258  1493  79722  151056  22806  51744  23569  3236  640  122618  3118  3798  7124  3557  3570  4982 | Arthritis, Rheumatoid  Arthritis, Rheumatoid  Arthritis, Rheumatoid  Arthritis, Rheumatoid  Arthritis, Rheumatoid  Arthritis, Rheumatoid  Arthritis, Rheumatoid  Arthritis, Rheumatoid  Arthritis, Rheumatoid  Arthritis, Rheumatoid  Arthritis, Rheumatoid  Arthritis, Rheumatoid  Arthritis, Rheumatoid  Arthritis, Rheumatoid  Arthritis, Rheumatoid  Arthritis, Rheumatoid  Arthritis, Rheumatoid  Arthritis, Rheumatoid  Arthritis, Rheumatoid  Arthritis, Rheumatoid  Arthritis, Rheumatoid  Arthritis, Rheumatoid  Arthritis, Rheumatoid  Arthritis, Rheumatoid  Arthritis, Rheumatoid  Arthritis, Rheumatoid  Arthritis, Rheumatoid  Arthritis, Rheumatoid  Arthritis, Rheumatoid  Arthritis, Rheumatoid  Arthritis, Rheumatoid  Arthritis, Rheumatoid  Arthritis, Rheumatoid  Arthritis, Rheumatoid  Arthritis, Rheumatoid  Arthritis, Rheumatoid  Arthritis, Rheumatoid  Arthritis, Rheumatoid  Arthritis, Rheumatoid  Arthritis, Rheumatoid  Arthritis, Rheumatoid  Arthritis, Rheumatoid  Arthritis, Rheumatoid  Arthritis, Rheumatoid  Arthritis, Rheumatoid  Arthritis, Rheumatoid  Arthritis, Rheumatoid  Arthritis, Rheumatoid  Arthritis, Rheumatoid  Arthritis, Rheumatoid  Arthritis, Rheumatoid  Arthritis, Rheumatoid  Arthritis, Rheumatoid  Arthritis, Rheumatoid  Arthritis, Rheumatoid  Arthritis, Rheumatoid  Arthritis, Rheumatoid  Arthritis, Rheumatoid  Arthritis, Rheumatoid  Arthritis, Rheumatoid  Arthritis, Rheumatoid  Arthritis, Rheumatoid  Arthritis, Rheumatoid  Arthritis, Rheumatoid  Arthritis, Rheumatoid  Arthritis, Rheumatoid  Arthritis, Rheumatoid  Arthritis, Rheumatoid  Arthritis, Rheumatoid  Arthritis, Rheumatoid  Arthritis, Rheumatoid  Arthritis, Rheumatoid  Arthritis, Rheumatoid  Arthritis, Rheumatoid  Arthritis, Rheumatoid  Arthritis, Rheumatoid  Arthritis, Rheumatoid  Arthritis, Rheumatoid  Arthritis, Rheumatoid  Arthritis, Rheumatoid  Arthritis, Rheumatoid  Arthritis, Rheumatoid  Arthritis, Rheumatoid  Arthritis, Rheumatoid  Arthritis, Rheumatoid  Arthritis, Rheumatoid  Arthritis, Rheumatoid  Arthritis, Rheumatoid  Arthritis, Rheumatoid  Arthritis, Rheumatoid  Arthritis, Rheumatoid  Arthritis, Rheumatoid  Arthritis, Rheumatoid  Arthritis, Rheumatoid  Arthritis, Rheumatoid  Arthritis, Rheumatoid  Arthritis, Rheumatoid  Arthritis, Rheumatoid  Arthritis, Rheumatoid  Arthritis, Rheumatoid  Arthritis, Rheumatoid  Arthritis, Rheumatoid  Arthritis, Rheumatoid  Arthritis, Rheumatoid  Arthritis, Rheumatoid  Arthritis, Rheumatoid  Arthritis, Rheumatoid  Arthritis, Rheumatoid  Arthritis, Rheumatoid  Arthritis, Rheumatoid  Arthritis, Rheumatoid  Arthritis, Rheumatoid  Arthritis, Rheumatoid  Arthritis, Rheumatoid  Arthritis, Rheumatoid  Arthritis, Rheumatoid  Arthritis, Rheumatoid  Arthritis, Rheumatoid  Arthritis, Rheumatoid  Arthritis, Rheumatoid  Arthritis, Rheumatoid  Arthritis, Rheumatoid  Arthritis, Rheumatoid  Arthritis, Rheumatoid  Arthritis, Rheumatoid  Arthritis, Rheumatoid  Arthritis, Rheumatoid  Arthritis, Rheumatoid  Arthritis, Rheumatoid  Arthritis, Rheumatoid  Arthritis, Rheumatoid  Arthritis, Rheumatoid  Arthritis, Rheumatoid  Arthritis, Rheumatoid  Arthritis, Rheumatoid  Arthritis, Rheumatoid  Arthritis, Rheumatoid  Arthritis, Rheumatoid  Arthritis, Rheumatoid  Arthritis, Rheumatoid  Arthritis, Rheumatoid  Arthritis, Rheumatoid  Arthritis, Rheumatoid  Arthritis, Rheumatoid  Arthritis, Rheumatoid  Arthritis, Rheumatoid  Arthritis, Rheumatoid  Arthritis, Rheumatoid  Arthritis, Rheumatoid  Arthritis, Rheumatoid  Arthritis, Rheumatoid  Arthritis, Rheumatoid  Arthritis, Rheumatoid  Arthritis, Rheumatoid  Arthritis, Rheumatoid  Arthritis, Rheumatoid  Arthritis, Rheumatoid  Arthritis, Rheumatoid  Arthritis, Rheumatoid  Arthritis, Rheumatoid  Arthritis, Rheumatoid  Arthritis, Rheumatoid  Arthritis, Rheumatoid  Arthritis, Rheumatoid  Arthritis, Rheumatoid  Arthritis, Rheumatoid  Arthritis, Rheumatoid  Arthritis, Rheumatoid  Arthritis, Rheumatoid  Arthritis, Rheumatoid  Arthritis, Rheumatoid  Arthritis, Rheumatoid  Arthritis, Rheumatoid  Arthritis, Rheumatoid  Arthritis, Rheumatoid | marker/mechanism  marker/mechanism  marker/mechanism  marker/mechanism  marker/mechanism  marker/mechanism  marker/mechanism  marker/mechanism  marker/mechanism  marker/mechanism  marker/mechanism  marker/mechanism  marker/mechanism  marker/mechanism  marker/mechanism  marker/mechanism  marker/mechanism  marker/mechanism  marker/mechanism  marker/mechanism  marker/mechanism  marker/mechanism  marker/mechanism  marker/mechanism  marker/mechanism  marker/mechanism  marker/mechanism  marker/mechanism  marker/mechanism  marker/mechanism  marker/mechanism  marker/mechanism  marker/mechanism  marker/mechanism  marker/mechanism  marker/mechanism  marker/mechanism  marker/mechanism  marker/mechanism  marker/mechanism  marker/mechanism  marker/mechanism  marker/mechanism  marker/mechanism  marker/mechanism  marker/mechanism  marker/mechanism  marker/mechanism  marker/mechanism  marker/mechanism  marker/mechanism  marker/mechanism  marker/mechanism  marker/mechanism  marker/mechanism  marker/mechanism  marker/mechanism  marker/mechanism  marker/mechanism  marker/mechanism  marker/mechanism  marker/mechanism  marker/mechanism  marker/mechanism  marker/mechanism  marker/mechanism  marker/mechanism  marker/mechanism  marker/mechanism  marker/mechanism  marker/mechanism  marker/mechanism  marker/mechanism  marker/mechanism  marker/mechanism  marker/mechanism  marker/mechanism  marker/mechanism  marker/mechanism  marker/mechanism  marker/mechanism  marker/mechanism  marker/mechanism  marker/mechanism  marker/mechanism  marker/mechanism  marker/mechanism  marker/mechanism  marker/mechanism  marker/mechanism  marker/mechanism  marker/mechanism  marker/mechanism  marker/mechanism  marker/mechanism  marker/mechanism  marker/mechanism  marker/mechanism  marker/mechanism  marker/mechanism  marker/mechanism  marker/mechanism  marker/mechanism  marker/mechanism  marker/mechanism  marker/mechanism  marker/mechanism  marker/mechanism  marker/mechanism  marker/mechanism  marker/mechanism  marker/mechanism  marker/mechanism  marker/mechanism  marker/mechanism  marker/mechanism  marker/mechanism  marker/mechanism  marker/mechanism  marker/mechanism  marker/mechanism  marker/mechanism  marker/mechanism  marker/mechanism  marker/mechanism  marker/mechanism  marker/mechanism  marker/mechanism  marker/mechanism  marker/mechanism  marker/mechanism  marker/mechanism  marker/mechanism  marker/mechanism  marker/mechanism  marker/mechanism  marker/mechanism  marker/mechanism  marker/mechanism  marker/mechanism  marker/mechanism  marker/mechanism  marker/mechanism  marker/mechanism  marker/mechanism  marker/mechanism  marker/mechanism  marker/mechanism  marker/mechanism  marker/mechanism  marker/mechanism  marker/mechanism  marker/mechanism  marker/mechanism  marker/mechanism  marker/mechanism  marker/mechanism  marker/mechanism  marker/mechanism  marker/mechanism  marker/mechanism  marker/mechanism  marker/mechanism  marker/mechanism  marker/mechanism  marker/mechanism  marker/mechanism  marker/mechanism  marker/mechanism  marker/mechanism  marker/mechanism  marker/mechanism|therapeutic  marker/mechanism|therapeutic  marker/mechanism|therapeutic  therapeutic |


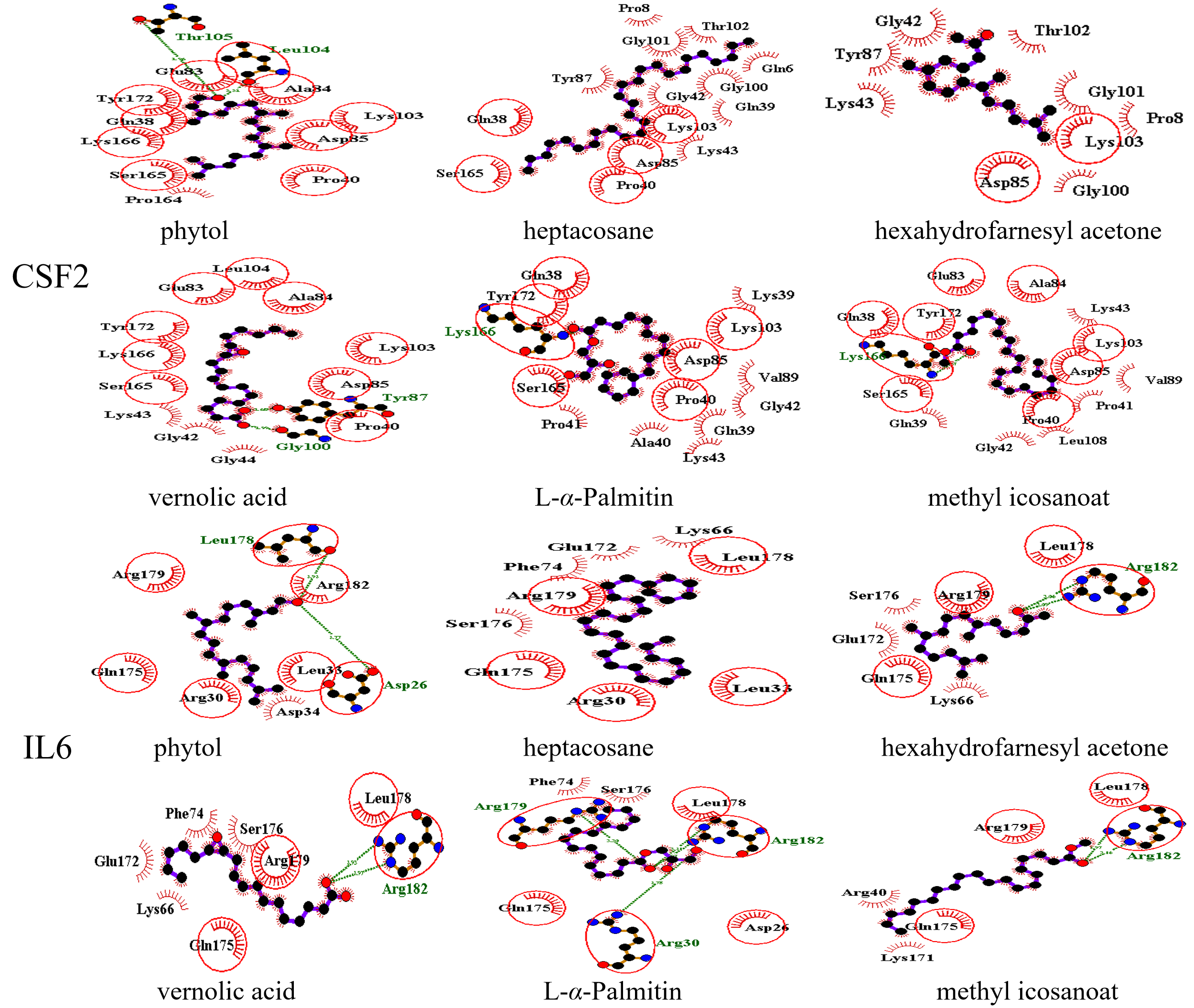


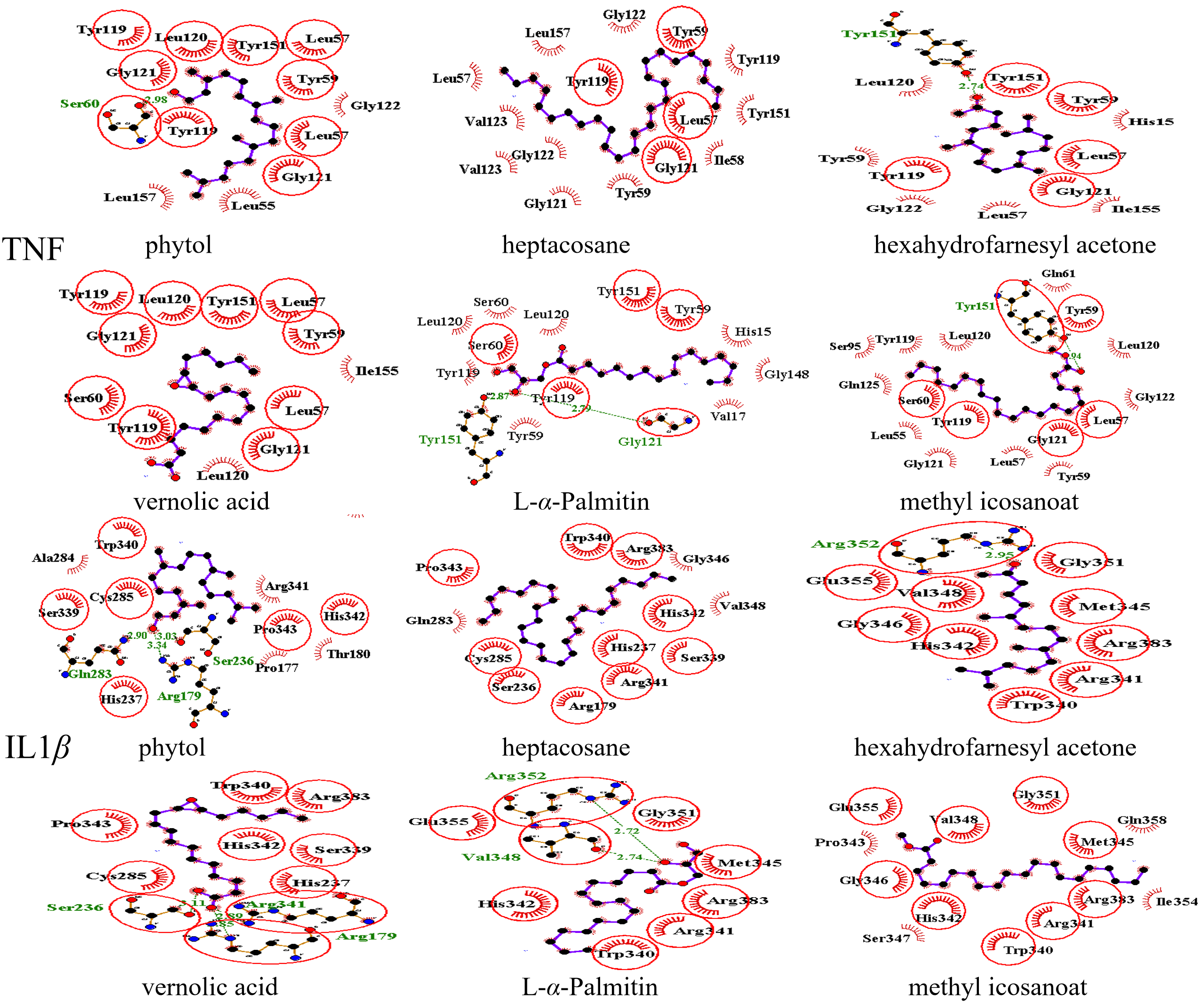


Figure S1: Structure of interaction between main components and target point
